# Early Effects of Single and Low-Frequency Repeated Administration of Teriparatide, hPTH(1-34), on Bone Formation and Resorption in Ovariectomized Rats

**DOI:** 10.1007/s00223-015-0026-1

**Published:** 2015-07-04

**Authors:** Yukihiro Isogai, Ryoko Takao-Kawabata, Aya Takakura, Emika Sugimoto, Osamu Nakazono, Ichiro Ikegaki, Hiroshi Kuriyama, Toshinori Ishizuya

**Affiliations:** Laboratory for Pharmacology, Pharmaceutical Research Center, Asahi Kasei Pharma Corporation, 632-1 Mifuku, Izunokuni-shi, Shizuoka 410-2321 Japan

**Keywords:** Teriparatide, Bone turnover marker, Bone formation, Bone resorption, Histomorphometry

## Abstract

Intermittent repeated administration of teriparatide (TPTD) has potent anabolic effects on bones in vivo. However, TPTD has both anabolic and catabolic effects on osteoblasts in vitro, and the mechanisms underlying its promotion of bone formation are unclear. This study aimed to elucidate the time-dependent changes in bone formation and resorption by examining changes in bone turnover markers and bone tissue over time after TPTD administration with low frequency in ovariectomized rats. The amount of serum osteocalcin, a bone formation marker, was transiently reduced after single TPTD administration, but increased thereafter, remaining increased for several days. In contrast, the amount of excreted urinary C-telopeptide, a bone resorption marker, increased transiently after single TPTD administration, and subsequently returned to control levels on the day after administration. Tissue histomorphometric analyses conducted 8 h after administration showed no changes in bone formation or bone resorption parameters. However, at 48 h, the bone formation parameters OS/BS and Ob.S/BS were increased, while the bone resorption parameter ES/BS was decreased. After repeated TPTD administration for 4 weeks, OS/BS, Ob.S/BS, and MS/BS increased, while Oc.S/BS decreased. Serum osteocalcin at 4 weeks after repeated administration was significantly correlated with OS/BS and Ob.S/BS. These present findings indicate that TPTD has dual, time-dependent effects on bone resorption and bone formation. Immediately after single administration, there was transient promotion of bone resorption and suppression of bone formation. However, sustained stimulation of bone formation occurred thereafter. Furthermore, these data suggest that this sustained bone formation led to anabolic effects with repeated TPTD administration.

## Introduction

Intermittent administration of teriparatide (TPTD) has been used in the treatment of severe osteoporosis [1]. Daily treatment with TPTD accelerates bone turnover, and bone formation markers such as P1CP and P1NP are elevated from the beginning of treatment [2, 3]. Bone resorption markers such as type I collagen N-telopeptide and type I collagen C-telopeptide (CTX) are also elevated several months after the beginning of treatment, and this is explained as the “anabolic window” of TPTD [4, 5].

Weekly treatment with TPTD is now being used clinically for the treatment of osteoporosis in Japan [6]. Although the effects of weekly TPTD treatment on bone mineral density and vertebral fractures are comparable to those of daily treatment [6, 7], its effects on bone turnover markers differ from those of daily treatment [2, 6]. Specifically, the increasing effects of weekly TPTD treatment on bone formation markers are relatively small compared with those of daily treatment, while bone resorption markers increase with daily TPTD treatment but tend to decrease with weekly TPTD treatment [2, 6]. These effects comprise a different phenomenon from the anabolic window that is evident with daily treatment, suggesting distinct effects on bone turnover depending on the frequency of TPTD administration. However, it remains unknown why such differences exist in bone turnover markers depending on the frequency of TPTD administration.

Controversial results have been reported regarding the effects of TPTD on bone formation. With repeated administration, bone formation markers are elevated regardless of the frequency of administration [2, 6]. However, in examinations of the time-dependent changes after single TPTD administration, bone formation markers have been shown to decrease immediately after administration [8–10].

Shiraki et al. [8] described the changes in bone turnover markers following single injection of 28.2 or 56.5 μg TPTD in healthy elderly women. They observed that single injection of TPTD caused an immediate, transient increase in bone resorption markers and decrease in bone formation markers, followed by increased bone formation markers and decreased bone resorption markers for at least 1 week. However, it remains unclear whether the changes in these markers accurately reflect the changes in bone tissue, and whether the changes resulting from single administration can also explain the changes that occur after repeated administration.

The aim of this study was to reveal the primary effects of TPTD administration on bone formation and resorption by evaluating bone turnover markers and bone histomorphometry in ovariectomized (OVX) rats. We also examined the effects of intermittent repeated TPTD administration on bone turnover markers and bone histomorphometric parameters.

## Methods

### Drug Used

Human parathyroid hormone PTH(1-34) (TPTD; Asahi Kasei Pharma Corporation, Shizuoka, Japan) was dispensed into vials, lyophilized, and dissolved in specific amounts of vehicle (saline) to create solutions with the target concentrations. The TPTD concentrations in the vials were analyzed by HPLC to confirm that the errors between before and after the study were within 10 %.

### Animals

Eleven-week-old female Sprague–Dawley rats (Charles River, Kanagawa, Japan) were maintained under a 12-h/12-h light/dark cycle with unrestricted access to tap water and a standard diet containing 1.2 % Ca, 0.9 % P, 22 % protein, and 6.2 IU vitamin D3 per gram (CRF-1; Oriental Yeast, Tokyo, Japan). Sham surgery or ovariectomy was performed at 13 weeks of age. All animal studies were reviewed before implementation by an internal animal welfare committee to ensure compliance with the NIH guidelines.

### Experimental Protocols

#### Outline

Six experiments were carried out in this study. First, the effects of single administration of TPTD on bone formation markers, bone resorption markers, and histomorphometric parameters were examined in three independent experiments. Second, the effects of repeated administration of TPTD on bone formation markers and histomorphometric parameters were examined in two independent experiments. Finally, the relationships between bone formation markers and histomorphometric bone formation parameters were examined in one independent experiment.

#### Single Administration Study for Bone Formation Markers

Thirty OVX rats were divided into three groups (*n* = 10) based on their serum osteocalcin (OC) levels at 14 days after surgery. A single subcutaneous administration of vehicle (saline) or TPTD (5.6 or 28.2 μg/kg) was performed at 16 days after surgery. Blood samples were collected from the cervical vein under diethyl ether anesthesia at 2 days before administration (pre), and at 6 h and 1, 2, 3, 4, and 7 days after administration.

#### Single Administration Study for Bone Resorption Markers

Twenty-four OVX rats were divided into two groups (*n* = 12) based on their urinary CTX levels at 23 days after surgery. The animals were fasted for more than 6 h before urine sample collection. A single subcutaneous administration of vehicle or TPTD (28.2 µg/kg) was performed at 25 days after surgery. Urine samples were collected at 2 days before administration (pre), and at 0 days (0–4 and 4–8 h), 1 day (24–28 h), and 2 days (48–52 h) after administration using metabolic cages. The samples at 0–4, 24–28, and 48–52 h were collected from 14:00 to 18:00 h. The samples at 4–8 h were collected from 18:00 to 22:00 h.

#### Single Administration Study for Histomorphometry

Thirty-two OVX rats were divided into four groups (*n* = 8) based on their body weights. A single subcutaneous administration of vehicle or TPTD (56.5 μg/kg) was performed at 14 days after surgery. Necropsies were carried out at 8 and 48 h after administration, and the right tibiae were collected. Histomorphometric analyses were performed for the secondary spongiosa of the tibial proximal metaphysis.

In this examination, we did not perform bone labeling, as the bones were isolated at very early times after drug administration. Therefore, we only measured static parameters.

#### Repeated Administration Study for Bone Formation Markers

Thirty-two OVX rats were divided into four groups (*n* = 8) based on their body weights. Low-frequency (three times weekly) subcutaneous administration of vehicle or TPTD (1.1, 5.6, or 28.2 µg/kg) was started at 2 weeks after surgery. The doses and regimen of TPTD were set as described previously [11, 12]. Blood samples were collected from the cervical vein under diethyl ether anesthesia before administration (0 days), and at 2, 5, 9, 14, 21, and 28 days after administration.

#### Repeated Administration Study for Bone Histomorphometry

Sixteen OVX rats were divided into two groups (*n* = 8) based on their body weights and serum OC levels. Three times weekly subcutaneous administration of vehicle or TPTD (5.6 µg/kg) was started at 2 weeks after surgery, and 10 mg/mL/kg calcein (Dojindo Laboratories, Kumamoto, Japan) was administered for bone labeling at 11 and 3 days before necropsy (labeling schedule: 1–7–1–2). Necropsies were carried out at 4 weeks and 2 days after the last administration and the right tibiae were isolated. Histomorphometric analyses were performed at the secondary spongiosa of the tibial proximal metaphysis.

#### Relationships Between Bone Formation Markers and Histomorphometric Bone Formation Parameters

Fifteen sham rats and forty-five OVX rats were divided into four groups (*n* = 15) based on their body weights. Three times weekly subcutaneous administration of vehicle or TPTD (5.6 or 28.2 μg/kg) was started at 2 weeks after surgery, and 20 mg/mL/kg tetracycline (Sigma-Aldrich, St. Louis, MO) and 10 mg/mL/kg calcein were administered for bone labeling at 8 and 3 days before necropsy (labeling schedule: 1–4–1–2). Blood samples were collected from the cervical vein under diethyl ether anesthesia before administration (0 days) and at 4 weeks after administration. Animals were euthanized at 4 weeks and 2 days after the last administration by exsanguination under anesthesia, and the fifth lumbar vertebrae were obtained.

### Measurement of Bone Turnover Markers

Serum OC levels were analyzed by ELISA (Rat Osteocalcin ELISA; GE Healthcare Bioscience, Tokyo, Japan). Urinary CTX concentrations were also analyzed by ELISA (RatLaps EIA; Immunodiagnostic Systems, Boldon, United Kingdom). Urinary creatinine (Cre) was measured by an enzymatic method (Pureauto S CRE-N; Sekisui Medical, Tokyo, Japan). The urinary CTX concentrations were corrected by the corresponding Cre concentrations.

### Histomorphometry

The right tibiae or fifth lumbar vertebrae were removed, dissected free of soft tissue, fixed in 70 % ethanol, stained with Villanueva bone stain, dehydrated in a graded ethanol series, defatted in acetone, and embedded in polymethyl methacrylate (Wako Pure Chemical Industries, Osaka, Japan). Thin sections (5 μm) were prepared from a sagittal section of the central fifth lumbar vertebrae and a coronal section of the proximal tibiae.

Bone histomorphometric parameters related to bone mass, structure, resorption, formation, and turnover were measured using an image analysis system (Histometry RT Camera; System Supply, Nagano, Japan). Histomorphometric measurements were obtained from cancellous bone tissue in the secondary spongiosa region. The secondary spongiosa region was defined as the interior of the fifth lumbar vertebra at 1.0 mm from the cranial and caudal growth plates and 0.5 mm from the cortical bone. Measurements were performed on half of the secondary spongiosa region on the caudal side.

Abbreviations for bone histomorphometric parameters are according to Dempster et al. [13]. The following static parameters were measured: trabecular bone volume (BV/TV); trabecular thickness (Tb.Th); trabecular number (Tb.N); and trabecular separation (Tb.Sp). The following bone formation parameters were measured: osteoid surface (OS/BS); osteoblast surface (Ob.S/BS); mineralizing surface (MS/BS); mineral apposition rate (MAR); and bone formation rate (BFR/BV). The following bone resorption parameters were measured: eroded surface (ES/BS); osteoclast surface (Oc.S/BS); osteoclast number (N.Oc/BS); and erosion depth (E.De). The following bone turnover parameter was measured: activation frequency (Ac.f).

### Statistical Analysis

All results are expressed as mean ± SD. Statistical analyses of differences among data were performed by a *t* test for two groups and by Dunnett’s test for multiple groups. The time-course studies were analyzed by analysis of variance (ANOVA). Significance was defined as values of *P* < 0.05.

## Results

### Bone Turnover Markers after Single Administration

**Fig. 1:**
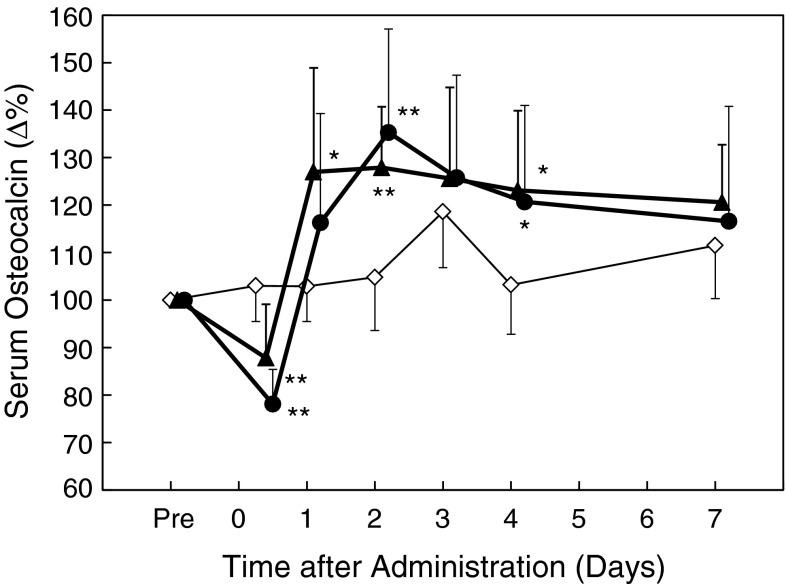
Early effects of single administration of teriparatide on serum osteocalcin levels in ovariectomized rats. The mean percent changes in serum osteocalcin during 7 days after single subcutaneous administration of teriparatide (*filled triangles*, 5.6 μg/kg; *filled circles*, 28.2 μg/kg) or vehicle (*empty diamonds*) are shown. Blood samples were collected at 2 days before administration (pre), and at 6 h (0 days) and 1, 2, 3, 4, and 7 days after administration. Data are shown as mean ± SD (*n* = 10). *P < 0.05, **P < 0.01, versus vehicle (Dunnett’s test)

The percent changes from the pretreatment values were calculated for serum OC. In the TPTD-treated groups, serum OC was transiently decreased by 15 and 13 % (for 5.6 and 28.2 μg/kg, respectively) at 6 h after administration, compared with that in the vehicle-treated group (Fig. 1). Thereafter, serum OC was elevated by 24 and 13 % at 1 day (24 h) and by 23 and 31 % at 2 days, compared with that in the vehicle-treated group, and these levels were maintained for several days, before returning to the vehicle-treated levels at 7 days after administration (Fig. 1). There were no obvious dose-dependent differences in the serum OC changes.

**Fig. 2:**
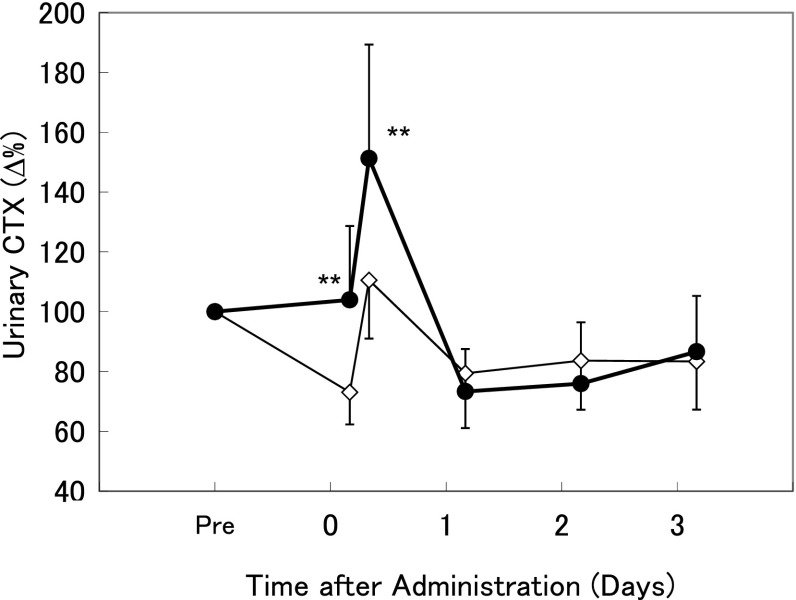
Early effects of single administration of teriparatide on urinary type I collagen C-telopeptide (CTX) in ovariectomized rats. The mean percent changes in urinary CTX during 3 days after single subcutaneous administration of 28.2 μg/kg teriparatide (*filled circles*) or vehicle (*empty diamonds*) are shown. Urine samples were collected at 2 days before administration (pre), and at 0 days (0–4 and 4–8 h), 1 day (24–28 h), and 2 days (48–52 h) after administration. Data are shown as mean ± SD (*n* = 12). **P < 0.01, versus vehicle (*t* test)

The percent changes from the pretreatment values were also calculated for urinary CTX, corrected by urinary Cre. In the vehicle-treated group on day 0, the value at 4–8 h was higher than the value at 0–4 h (Fig. 2). In the TPTD-treated group, urinary CTX was increased by 31 % at 0–4 h and 41 % at 4–8 h compared with that in the vehicle-treated group. There were no differences between the TPTD-treated and vehicle-treated groups from day 1 to day 3.

### Bone Histomorphometric Parameters After Single Administration

At 8 h after administration, the histomorphometric parameters did not differ between the vehicle-treated and TPTD-treated groups (Table 1). E.De increased slightly, but not significantly, in the TPTD-treated group. At 48 h after administration, Ob.S/BS had increased by 56 %, OS/BS had increased by 46 %, and ES/BS had decreased by 14 % in the TPTD-treated group.

**Table 1:** Early effects of single administration of teriparatide on bone histomorphometric parameters in ovariectomized rats

Parameters (unit)	8 h			48 h		
	Vehicle	Teriparatide	*P*	Vehicle	Teriparatide	*P*
	Mean ± SD	Mean ± SD		Mean ± SD	Mean ± SD	
BV/TV (%)	16.805 ± 3.123	15.370 ± 5.197	0.514	15.673 ± 3.26	17.683 ± 4.561	0.328
Tb.Th (µm)	44.064 ± 4.142	40.432 ± 2.562	0.053	46.357 ± 5.831	47.038 ± 3.293	0.778
Tb.N (*N*/mm)	3.826 ± 0.727	3.766 ± 1.153	0.901	3.373 ± 0.471	3.741 ± 0.832	0.295
Tb.Sp (µm)	225.52 ± 49.21	257.23 ± 127.65	0.523	255.78 ± 47.89	233.39 ± 70.64	0.470
OS/BS (%)	15.574 ± 4.732	14.203 ± 8.867	0.705	23.074 ± 4.441	33.776 ± 5.079	0.001
Ob.S/BS (%)	13.081 ± 3.877	11.827 ± 6.933	0.662	20.884 ± 1.818	32.638 ± 4.460	<0.001
ES/BS (%)	32.361 ± 6.996	30.387 ± 8.016	0.608	37.565 ± 2.972	32.393 ± 2.302	0.002
Oc.S/BS (%)	14.944 ± 3.954	14.249 ± 4.851	0.758	16.564 ± 1.769	15.007 ± 1.676	0.092
E.De (µm)	2.839 ± 0.717	3.313 ± 0.232	0.097	ND	ND	

Tibiae were isolated at 8 and 48 h after single subcutaneous administration of vehicle or 56.5 μg/kg teriparatide. Histomorphometric analyses were performed at the secondary spongiosa of the tibial proximal metaphysis. Data are shown as mean ± SD (*n* = 8)

*ND* not determined, *BV/TV* trabecular bone volume, *Tb.Th* trabecular thickness, *Tb.N* trabecular number, *Tb.Sp* trabecular separation, *OS/BS* osteoid surface, *Ob.S/BS* osteoblast surface, *ES/BS* eroded surface, *Oc.S/BS* osteoclast surface, *E.De* erosion depth

*P* values are shown for comparisons between the vehicle-treated group and the teriparatide-treated group at the same time points (*t* test)

### Bone Formation Markers After Repeated Administration

**Fig. 3:**
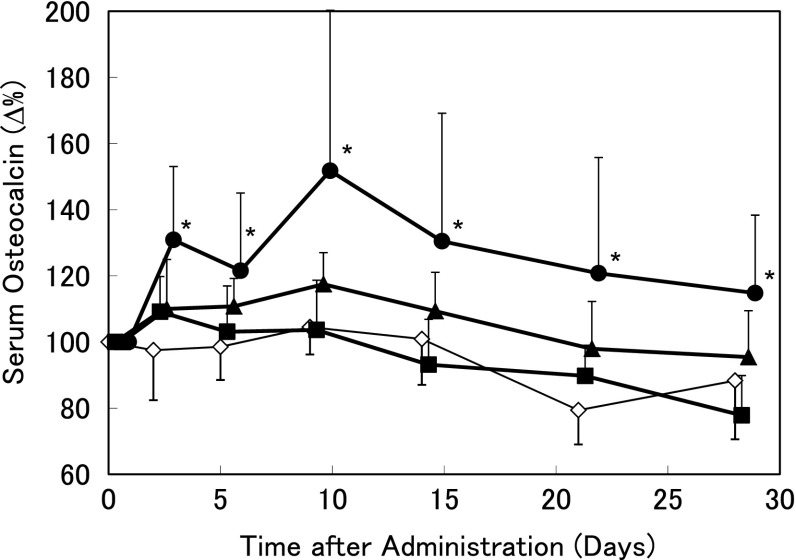
Early effects of repeated administration of teriparatide on serum osteocalcin levels in ovariectomized rats. The mean percent changes in serum osteocalcin during 28 days after three times weekly subcutaneous administration of teriparatide (*filled squares*, 1.1 μg/kg; *filled triangles*, 5.6 μg/kg; *filled circles*, 28.2 μg/kg) or vehicle (*empty diamonds*) are shown. Blood samples were collected just before administration (0 days), and at 2, 5, 9, 14, 21, and 28 days after administration. Data are shown as mean ± SD (*n* = 8). *P < 0.05, versus vehicle (Dunnett’s test)

TPTD increased serum OC by intermittent repeated administration (three times weekly) in OVX rats (Fig. 3). Serum OC peaked at day 2 and showed sustained dose-dependent increases compared with the levels in the vehicle-treated group of about 10 % for 5.6 μg/kg TPTD and 30 % for 28.2 μg/kg TPTD.

### Bone Histomorphometric Parameters After Repeated Administration

Repeated injections of 5.6 µg/kg TPTD for 4 weeks increased trabecular BV/TV by 39 % and Tb.Th by 18 % in OVX rats compared with those in the vehicle-treated group (Table 2). The bone formation parameters Ob.S/BS, OS/BS, and MS/BS increased by 74, 76, and 43 % in the TPTD-treated group, respectively. Meanwhile, the bone resorption parameter Oc.S/BS decreased by 12 % in the TPTD-treated group.

**Table 2:** Effects of repeated administration of teriparatide on bone histomorphometric parameters in ovariectomized rats

Parameters (units)	4 Weeks		
	Vehicle	Teriparatide	*P*
	Mean ± SD	Mean ± SD	
BV/TV (%)	17.335 ± 4.937	24.181 ± 7.478	0.049
Tb.Th (µm)	53.812 ± 5.374	63.615 ± 8.330	0.014
Tb.N (*N*/mm)	3.203 ± 0.746	3.744 ± 0.825	0.191
Tb.Sp (µm)	273.09 ± 75.79	218.47 ± 84.01	0.194
OS/BS (%)	18.278 ± 6.010	32.077 ± 8.095	0.002
Ob.S/BS (%)	14.263 ± 2.839	24.789 ± 5.191	<0.001
ES/BS (%)	26.335 ± 4.291	24.273 ± 3.453	0.308
Oc.S/BS (%)	13.493 ± 2.013	11.822 ± 2.318	0.038
MS/BS (%)	27.837 ± 7.542	39.863 ± 6.637	0.004
MAR (µm/day)	1.067 ± 0.168	1.244 ± 0.212	0.084
BFR/BV (%/year)	413.96 ± 161.06	574.54 ± 139.08	0.051
Ac.f (*N*/year)	5.943 ± 2.389	9.208 ± 2.927	0.028

Tibiae were isolated after three times weekly subcutaneous administration of vehicle or 5.6 µg/kg teriparatide for 4 weeks. Histomorphometric analyses were performed at the secondary spongiosa of the tibial proximal metaphysis. Data are shown as mean ± SD (*n* = 8)

*BV/TV* trabecular bone volume, *Tb.Th* trabecular thickness, *Tb.N* trabecular number, *Tb.Sp* trabecular separation, *OS/BS* osteoid surface, *Ob.S/BS* osteoblast surface, *ES/BS* eroded surface, *Oc.S/BS* osteoclast surface, *MS/BS* mineralizing surface, *MAR* mineral apposition rate, *BFR/BV* bone formation rate, *Ac.f* activation frequency

*P* values are shown for comparisons between the vehicle-treated group and the teriparatide-treated group (*t* test)

### Relationships Between Bone Formation Marker and Histomorphometric Bone Formation Parameters

**Fig. 4:**
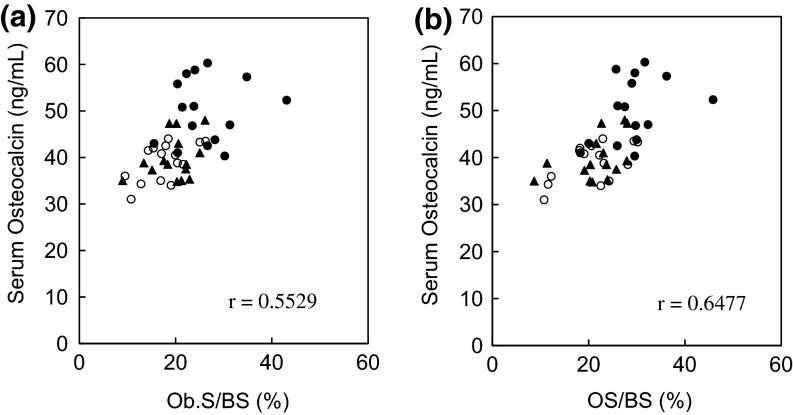
Correlations between histomorphometric bone formation parameters and serum osteocalcin levels during repeated administration of teriparatide in ovariectomized rats. The correlations between Ob.S/BS and serum osteocalcin (**a**) and between OS/BS and serum osteocalcin (**b**) at 4 weeks after three times weekly subcutaneous administration of teriparatide (*filled triangles*, 5.6 μg/kg; *filled circles*, 28.2 μg/kg) or vehicle (*empty circles*) in ovariectomized rats are shown. The histomorphometric analyses were performed at the secondary spongiosa of the lumbar vertebrae. A total of 15 data points are plotted for each group

After 4 weeks of administration, serum OC was significantly higher in the OVX group (39.0 ± 4.1 ng/mL) than in the Sham group (29.7 ± 1.9 ng/mL). TPTD dose dependently increased serum OC to 39.8 ± 4.6 ng/mL at a dose of 5.6 μg/kg and 49.9 ± 7.0 ng/mL at a dose of 28.2 μg/kg. The histomorphometric bone formation parameters of Ob.S/BS and OS/BS in the lumbar spine were also significantly increased in a dose-dependent manner in the OVX group compared with those in the control group. Ob.S/BS and OS/BS were correlated with serum OC at 4 weeks (Pearson’s correlation coefficient, *r* = 0.5529 and *r* = 0.6477, respectively, Fig. 4).

## Discussion

The aim of this study was to assess the primary effects of TPTD administration on bone formation and resorption by evaluating bone turnover markers and bone histomorphometric parameters in OVX rats. We assessed the changes in bone turnover markers over time after single TPTD injection. The bone formation marker serum OC transiently decreased at 6 h after administration, subsequently increased after 24 h, and returned to the control levels after 7 days. In addition, the bone resorption marker urinary CTX increased transiently up to 8 h after administration, but then returned to the vehicle-treated levels after 24 h. The bone formation and resorption dynamics were analyzed by bone histomorphometry after single TPTD administration. The bone formation parameters Ob.S/BS and OS/BS were significantly elevated at 48 h after administration. These observations suggest that TPTD promotes bone formation by increasing osteoblasts, thereby increasing the bone formation marker serum OC. In contrast, TPTD administration resulted in a decreasing trend for the bone resorption parameter Oc.S/BS and significantly decreased ES/BS. These findings indicate that TPTD decreases bone resorption by decreasing the number and activity of osteoclasts.

It is recognized that OC is a bone remodeling (or turnover) marker produced by osteoblasts and also released from the bone matrix by osteoclasts. Therefore, changes in serum OC reflect not only bone formation, but also bone resorption. In this study, a bone resorption marker was increased for several hours after single administration of TPTD. At the same time, serum OC was decreased. This indicated that serum OC was not overestimated by the increase in serum OC induced by bone resorption. In addition, the bone resorption marker CTX returned to the baseline level within 24 h after single administration of TPTD (Fig. 2), and the histomorphometric resorption parameter was not increased after three times weekly repeated administration (Table 2). Therefore, we consider that the serum OC levels after single administration of TPTD (Fig. 1) and repeated administration (Fig. 3) reflect bone formation activity.

Serum P1NP is considered to be a good bone formation marker that enables accurate conclusions to be made in humans. In a preliminary study, we examined the effects of single and low-frequency (three times weekly) repeated administration of TPTD on serum OC and P1NP in OVX rats. We found that these administrations did not increase serum P1NP, but did increase serum OC and histomorphometric bone formation parameters. These preliminary results indicated that serum P1NP is not sufficiently validated for use as a bone formation marker in rats. We also examined bone-specific alkaline phosphatase (BAP) using an ELISA kit, but were unable to measure BAP in rat serum samples.

Meanwhile, no significant changes in trabecular BV/TV and the bone structure parameters Tb.Th, Tb.N, and Tb.Sp were observed. These observations indicate that the time period of 48 h after single TPTD administration does not allow sufficient time to induce changes in bone mass or bone structure, and that accumulation of TPTD through repeated administration is necessary to exert these effects.

In the present study, although serum OC decreased at 6 h after TPTD administration, tissue Ob.S/BS did not decrease after 8 h. Given that TPTD was shown to suppress osteoblast functions such as collagen synthesis and calcification in in vitro studies [14–17], it was considered that TPTD suppressed osteoblast functions without decreasing the osteoblast number. Although increases in bone resorption markers were observed at 4–8 h after administration, bone histomorphometric analyses conducted at 8 h after administration did not show any significant changes in the bone resorption parameters Oc.S/BS and ES/BS. Although E.De can only be measured by estimating the bone surface prior to osteoclast-induced bone erosion, and it cannot be measured with high precision, an increasing trend in E.De was nonetheless observed. Therefore, TPTD may have increased CTX by enhancing the bone resorption capacity of existing osteoclasts without affecting the number of osteoclasts at 8 h after administration. Another possibility is that CTX increased via osteocytic osteolysis [18].

Shiraki et al. [8] observed that a single injection of TPTD in postmenopausal women caused an immediate, transient decrease in serum OC for 2–3 days, followed by increased serum OC for at least 1 week. The duration of the serum OC decrease immediately after TPTD administration, as well as the subsequent duration of its increase, was both shorter in rats than in humans. The rapid changes in bone formation markers in rats were considered to arise because rats exhibit remodeling that is several times faster than that in humans [19–25]. In contrast, it has been reported that bone resorption markers increase up to 24 h after administration, and subsequently decline to significantly lower levels than in controls in humans [8], which is generally consistent with the changes in bone resorption markers observed in our rat study.

PTH is reported to increase RANKL expression on osteoblast lineage cells and to trigger osteoclast differentiation and activation. Ma et al. [26] reported that RANKL mRNA increased and osteoprotegerin mRNA decreased at 1 h after PTH administration in mice, but subsequently returned to the baseline levels. These responses after TPTD injection, in which bone resorption increased transiently and then returned to the basal levels after 24 h, were also confirmed in the present study.

Meanwhile, PTH in vitro has been reported to inhibit collagen synthesis [14], OC production [15], and calcified bone-like nodule formation in primary osteoblast cultures [15–17]. However, we [15] and Bellows et al. [16] found that removal of PTH from the culture medium leads to restoration of osteoblast function. In addition, PTH stimulates the proliferation and differentiation of osteoprogenitor cells and pre-osteoblasts [15, 27, 28], inhibits apoptosis [29, 30], and acts to gradually increase the osteoblast number.

Based on these findings, the 24-h responses in serum OC after injection of TPTD can be explained by inhibition of bone formation while TPTD is present in the blood, and the subsequent restoration of osteoblast function by elimination of TPTD from the blood. Moreover, the number of osteoblasts had increased by 48 h after administration, indicating that there was greater bone formation at this time point than there was pre-administration. Furthermore, with repeated administration, the cycle of short-term inhibition of bone formation and subsequent long-term stimulation of bone formation was repeated, and this was thought to increase the bone mass. When TPTD was administered daily, long-term stimulation of bone formation and short-term stimulation of bone resorption accumulated, resulting in marked increases in bone formation and resorption [2]. Meanwhile, TPTD administered weekly caused only mild accumulation of long-term stimulation of bone formation, and did not accumulate any short-term stimulation of bone resorption, resulting in a small increase of bone formation, but not bone resorption [6].

Single and repeated TPTD administration resulted in decreases in the bone resorption parameters Oc.S/BS and ES/BS. Shiraki et al. [8] reported that single administration of TPTD resulted in a decrease in endogenous intact PTH over 6 days in humans. Therefore, TPTD stimulated bone resorption for several hours immediately after its administration, but subsequently inhibited it for several days via endogenous PTH depression. This suggests that daily TPTD treatment increased bone resorption by continuing the stimulating effect on bone resorption every day [2], while weekly TPTD treatment induced phasic bone resorption at the administration day and prolonged decreased bone resorption by endogenous PTH depression thereafter [6]. The bone resorption parameters in the repeated administration study for bone histomorphometry were decreased because the bone samples were isolated at 2 days after the last administration.

In the present study, we showed correlations between bone formation markers assessed at 4 weeks after repeated TPTD administration and bone formation parameters determined by bone histomorphometric analyses. Although a few reports have previously described correlations between bone formation markers and tissue bone formation parameters [31–33], serum OC is considered to be a good index that reflects the bone formation status of bone tissue.

The present study has some limitations. The histomorphometric dynamic changes at 8 h after administration could not be determined. Therefore, conventional histomorphometry was considered to be insufficient to establish the short-term changes. TPTD is used in patients with severe osteoporosis, whereas relatively young OVX rats were used in this study.

In summary, immediately after single TPTD administration, bone resorption was promoted through transient osteoclast activation and bone formation was suppressed through inhibition of osteoblast function. However, after TPTD disappeared from the blood, the osteoblast number increased, resulting in increased bone formation. Furthermore, it is suggested that repeated TPTD administration induced an anabolic effect through the accumulation of this prolonged bone formation. Finally, it was demonstrated that serum OC is a good index for bone formation induced by TPTD.
